# Platelet, monocyte and neutrophil activation and glucose tolerance in South African Mixed Ancestry individuals

**DOI:** 10.1038/srep40329

**Published:** 2017-01-16

**Authors:** Glenda M. Davison, Bongani B. Nkambule, Zibusiso Mkandla, Gloudina M. Hon, Andre P. Kengne, Rajiv T. Erasmus, Tandi E. Matsha

**Affiliations:** 1 Department of Biomedical sciences, Faculty of Health and Wellness Sciences, Cape Peninsula University of Technology, Bellville, South Africa; 2 Department of Pathology, Faculty of Medicine and Health Sciences, National Health Laboratory Service (NHLS) and Stellenbosch University, Cape Town, South Africa; 3 NonCommunicable Diseases Research Unit, South African Medical Research Council, Cape Town, South Africa; 4 Department of Medicine, University of Cape Town, Cape Town, South Africa

## Abstract

Platelet activation has been described in patients with chronic inflammation, however in type 2 diabetes mellitus it remains controversial. We compared levels of platelet leucocyte aggregates, monocyte and granulocyte activation across glucose tolerance statuses in mixed ancestry South Africans. Individuals (206) were recruited from Bellville-South, Cape Town, and included 66% with normal glucose tolerance, 18.7% pre-diabetes, 8.7% screen-detected diabetes and 6.3% known diabetes. Monocyte and neutrophil activation were measured by calculating the percentage of cells expressing CD142 and CD69 while platelet monocyte aggregates were defined as CD14^++^ CD42b^+^ events and platelet neutrophil aggregates as CD16^++^ CD42b^+^ events. The percentage of monocytes and neutrophils expressing CD69 and CD142 was significantly higher in known diabetes and prediabetes, but, lowest in screen-detected diabetes (both p ≤ 0.016). The pattern was similar for platelet monocyte and neutrophil aggregates (both p ≤ 0.003). In robust linear regressions adjusted for age and gender, known diabetes was significantly and positively associated with the percentage of monocytes expressing CD69 [beta 11.06 (p = 0.016)] and CD42b (PMAs) [19.51 (0.003)] as well as the percentage of neutrophils expressing CD69 [14.19 (<0.0001)] and CD42b [17.7 (0.001)]. We conclude that monitoring platelet activation in diagnosed diabetic patients may have a role in the management and risk stratification.

Type 2 diabetes mellitus (T2D) is a metabolic disorder which is characterised by insulin resistance, defective insulin secretion or both. The consequent chronic state of hyperglycaemia is associated with chronic inflammation and atherothrombotic complications. It is thought that the pro-inflammatory environment leads to the vascular endothelial surface attracting both platelets and leucocytes which become activated, bind to the extracellular matrix and play a major role in the development of plaques and pathological thrombosis^1^.

Hyperinsulinaemia is a key pathogenic feature of T2D and both insulin and glucose has a direct effect on platelet function. It has been reported that glucose induces platelet hyperactivity via direct effects on cellular osmolality^2^,^3^ and activation of the protein kinase C (PKC) transduction pathway^4^. On the other hand, while insulin binds to the insulin receptor (IR) and inhibits platelet activation in normal individuals, recent research has suggested that in patients with T2D, platelets have reduced expression of the receptor and appear to be unable to respond to insulin^5^. Therefore although further research is needed, this could offer a further explanation for the hyperactivity, increased adhesiveness and responsiveness of platelets in T2D^6^,^7^,^8^.

Activated platelets play a key role in the initiation of both inflammation and coagulation^9^. Upon activation platelets degranulate and express a repertoire of membrane receptors which enable them to bind to circulating leukocytes via P-selectin^6^. P-selectin mediated interactions in turn activate leukocyte signal transduction pathways^10^ and initiate the rapid formation of platelet leukocyte aggregates (PLAs)^11^. Activated platelets preferentially bind to monocytes and form platelet monocyte aggregates (PMAs)^11^,^12^ which are a more sensitive and robust marker of platelet activation than the expression of P-selectin^13^,^14^.

Elevated circulating PMAs and PLAs have been described as an early marker of T2D^15^ and have also been reported in association with thrombotic^16^ and inflammatory conditions^13^,^17^. Importantly both PMA’s and PLA’s have been associated with vascular damage^18^. Although platelet activation has been described in patients with chronic inflammation, the presence of increased PMA’s in T2D remains controversial and recent research has shown that high risk T2D patients have normal functioning platelets with no increase in PMA’s or PLA’s^19^.

Therefore, although there is growing evidence that platelets and cells of the innate immune system are involved in the process of chronic inflammation and cardiovascular disease, their role in type 2 diabetes remains unclear. This study therefore aimed to investigate this issue by assessing the activation of neutrophils and monocytes. In addition, platelet activation was assessed by the measurement of platelet leukocyte aggregates across the spectrum of glucose tolerance in South African mixed ancestry individuals.

## Materials and Methods

### Ethical approval of the study

This investigation is based on the Bellville South (Ward 009) study^20^ from Cape Town that has been approved by the Research Ethics Committees of the Cape Peninsula University of Technology (CPUT) and Stellenbosch University (respectively, NHREC: REC - 230 408–014 and N14/01/003). For this sub-study, ethical approval was also obtained from the CPUT Health and Wellness Sciences Research Ethics Committee (CPUT/HW-REC 2014/H07). The study was conducted according to the Code of Ethics of the World Medical Association (Declaration of Helsinki). All participants signed written informed consent after all the procedures had been fully explained in the language of their choice.

### Study design and procedures

This was a cross-sectional study involving participants from the ongoing Cape Town Vascular and Metabolic Health (VMH) study. VMH is an extension of the Cape Town Bellville South study, which has been described in detail previously^20^. Participants were eligible for this study if they had undergone an overnight fast and were not on aspirin or anti-inflammatory drugs for a minimum of 14 days prior to sampling. In addition, participants were excluded if they showed clinical signs of recent infection, were pregnant or using immunosuppressant drugs. Data collection was based on a standardised questionnaire which included questions regarding the smoking status of the participant and was available in the electronic form on a password-protected personal digital assistant (PDA). Upon completion of the assessment of each participant, data were automatically encrypted and transmitted via mobile internet connection to a dedicated server, from which they were checked for completion, downloaded and stored for future use. Physical examination involved data collection on blood pressure (BP) which was measured according to the World Health Organisation (WHO) guidelines^21^, using a semi-automatic digital blood pressure monitor (Omron M6 comfort-preformed cuff BP Monitor) on the right arm in sitting position and at rest for at least 10 minutes. Body weight (to the nearest 0.1 kg) was measured with the subject in light clothing and without shoes, using an Omron body fat meter HBF-511digital bathroom scale, which also measures visceral fat, body fat percent and resting metabolic rate (RMR). Height to the nearest centimetre was measured with a stadiometer, with subjects standing on a flat surface. Body Mass Index (BMI) was calculated as weight per square meter (kg/m^2^). Waist circumference was measured with a non-elastic tape at the level of the narrowest part of the torso, as seen from the anterior view. Blood Pressure (BP) and anthropometric measurements were performed three times and their average used for analysis.

### Biochemical Assays

Blood samples were collected from all participants following an overnight fast. A 2-hour sample was obtained after a 75 g oral glucose tolerance test (OGTT) in participants with no history of doctor diagnosed diabetes mellitus. Routine biochemical parameters were analyzed at an ISO 15189 accredited Pathology practice (PathCare, Reference Laboratory, Cape Town, South Africa). Blood glucose (mmol/L) was measured using the enzymatic hexokinase method (Beckman AU, Beckman Coulter, South Africa). Insulin (mmol/L) was determined by a paramagnetic particle chemiluminescence assay (Beckman DXI, Beckman Coulter, South Africa). HDL-C (mmol/L) was by enzymatic immunoinhibition – End Point (Beckman AU, Beckman Coulter, South Africa). Low-density lipoprotein cholesterol (LDL-C) (mmol/L) was measured by enzymatic selective protection – End Point (Beckman AU, Beckman Coulter, South Africa). Triglycerides (TG) (mmol/L) were estimated by glycerol phosphate oxidase-peroxidase, End Point (Beckman AU, Beckman Coulter, South Africa). Glycated haemoglobin (HbA1c) was measured by High Perfomance Liquid Chromotography (Biorad Variant Turbo, BioRad, South Africa). Ultra sensitive C-reactive protein (U-CRP) was by Latex Particle immunoturbidimetric and the liver enzymes γ-Glutamyltransferase (GGT), alanine aminotransferase (ALT) and aspartate transaminase (AST) were measured using International Federation of Clinical Chemistry and Laboratory Medicine (IFCC) standardized reagents on a Beckman AU (Beckman Coulter, South Africa). Serum cotinine was measured by Competitive Chemiluminescent on Immulite 2000 (Siemens, Southern Africa). Full blood counts and platelets were measured on a Coulter LH 750 hematology analyser (Beckman Coulter, South Africa).

History, fasting glucose, and 2-hour glucose following OGTT were used to group participants for glucose tolerance status as normotolerant, prediabetes (including impaired fasting glycaemia, impaired glucose tolerance or the combination of both), screen-detected diabetes, and known diabetes, following the WHO criteria^22^. Cotinine levels were used to define smoking status based on a threshold of >50 ng/ml to characterise current smoking^23^.

### Flow cytometry

A volume of 2–3 ml of venous blood was collected into 4.5 ml tubes containing 3.2% sodium citrate (BD Vacutainer, San Jose, CA). To minimise artefactual activation due to tissue factor contamination, the first blood sample drawn was not used for flow cytometry. Measurements of platelet leukocyte aggregates were performed using fresh blood samples stained within 10 minutes of collection and analysed immediately to minimise the effects of time-dependent platelet and leukocyte activation kinetics^24^. This was made possible by the close proximity of the research clinic and the laboratory, both situated in the Department of Biomedical Sciences, Faculty of Health and Wellness Sciences, Cape Peninsula University of Technology.

**Figure 1:**
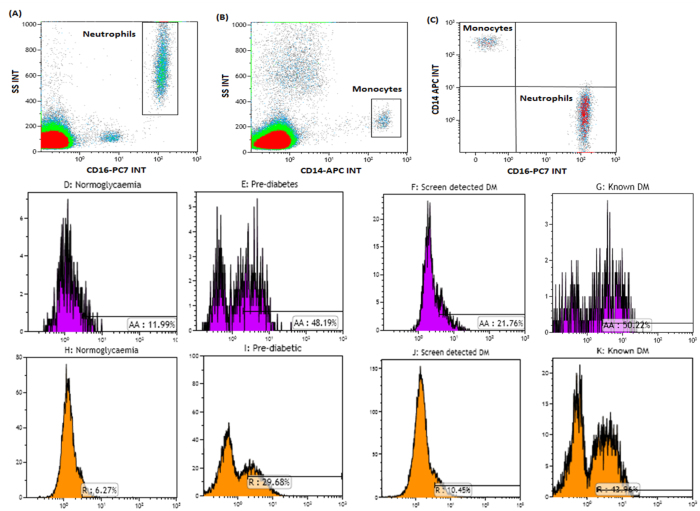
Gating strategy used to measure PMAs and PNAs. Classical monocytes and neutrophils expressing CD42b were regarded as PMAs and PNAs respectively. CD16^++^ neutrophils and CD14^++^ monocytes were gated as shown in (**A** and **B**). To further ensure that only classical monocytes (CD14^++^ CD16^−^) and unbound neutrophils (CD16^++^ CD14^−^) were analysed, the Boolean gate (Neutrophil “OR” Monocyte) was applied (shown in **C**). The expression of CD42b was then measured on both classical monocytes (**D**–**G**) and classical neutrophils (**H**–**K**).

The anti-human CD42b-FITC (SZ2); CD69-PC5 (TP1.55.3), CD14 APC (RM052), CD16-PC7 (3G8) antibodies were all purchased from Beckman Coulter, USA while anti-human CD142-PE (NY2) was purchased from Biolegend Inc, San Diego, CA. A multi-colour panel was set up to measure the levels of monocyte and neutrophil activation in peripheral whole blood. The flow cytometric measurement of PMAs and PNAs was performed as previously described^25^. Briefly, 50 μl of citrated whole blood was stained using 5 μl of the titrated monoclonal antibody cocktail and incubated for 15 minutes at room temperature. Red blood cell lysis was performed by incubating the samples with 500 μl FACSlyse (BD Biosciences, San Jose, CA) and thereafter diluted with 500 μl of PBS (without calcium or magnesium chloride). The stained cells were processed immediately using the Navios cytometer and analysed with the Kaluza V 1.3 analysis software (Beckman Coulter, USA) using the gating strategy shown in Fig. 1. Bright CD14^++^ monocytes and CD16^++^ neutrophils were gated (Fig. 1A,B). A Boolean gate was created (using the logic; Neutrophil “OR” Monocyte) and applied to ensure the gating of classical monocytes (CD14^++^ CD16^−^) and unbound neutrophils (CD16^++^ CD14^−^) (shown in Fig. 1C). FMO controls were used to distinguish tissue factor (CD142), CD69 (a marker of activation) positive and negative events.

The expression of CD42b on CD14^++^ monocytes and CD16^++^ neutrophils was assessed (Fig. 1D–K). An FMO control was used to distinguish between platelet bound (CD42b^+^) and unbound (CD42b^−^) monocytes and neutrophils. Platelet monocyte aggregates were defined as CD14^++^ CD42b^+^ events (Fig. 1D–G) and platelet neutrophil aggregates were defined as CD16^++^ CD42b^+^ events (Fig. 1H–K). A minimum of 2000 CD14+ events at a medium flow rate were counted for the analysis of platelet leukocyte aggregates. The mean fluorescent intensity (MFI) of expression was recorded as the geometric mean and was obtained from the relevant flow cytometric histogram using Kaluza v1.3 analysis software.

### Quality control measures

Daily cleaning of the flow cell fluidics line was performed using filtered Isoflo and Clenz^®^ (Beckman Coulter, USA). For daily monitoring of instrument performance, flow set beads (Beckman Coulter, USA) and flow check pro beads (Beckman Coulter, USA) were used and plotted on a levy Jennings plot. Antibody titrations were performed to determine the optimal antibody concentrations and colour compensation was performed using the versa comp antibody capture beads (Beckman Coulter, USA).

### Statistical analysis

Data were analysed with the used of R statistical software version 3.2.2 [2015-08-14] (The R Foundation for Statistical Computing Platform, Austria). Categorical variables are summarised as count and percentages and quantitative variables as mean (standard deviation) or median (25^th^–75^th^ percentiles). Variable comparisons across glucose tolerance statuses used chi square test, Analysis of the Variance (ANOVA) and Kruskal-Walis tests. The effects of glucose tolerance status and other predictors on platelet leucocyte aggregates, monocyte and granulocyte activation, was investigated using robust linear regressions models. Unlike conventional least squares based linear regressions, robust regressions are not sensitive to the effects of outliers which can be common with the outcome variables of interest in the current study, or when dealing with relatively small groups of participants. A p-value < 0.05 was used to characterise statistically significant results.

## Results

### General characteristics of the participants

The study sample comprised of 206 participants which included 136 (66%) with normal glucose tolerance, 39 (18.7%) with prediabetes (impaired fasting glycaemia and/or glucose tolerance), 18 (8.7%) with screen-detected diabetes and 13 (6.3%) with known diabetes all receiving at least metformin therapy (Table 1). The overall mean age was 52.2 (SD = 14.0) years, with a significant variation in glucose tolerance status (p = 0.007). One hundred and forty seven (71.4%) participants were female while eighty eight (42.9%) were smokers. There was no significant difference of both attributes across glucose tolerance status (both p > 0.103). Overall, the mean values (SD) for anthropometric measurements were as follows: body mass index (BMI) 29.5 (8.1) kg/m^2^, waist circumference 95 (15) cm, hip circumference 108 (15) cm, waist to hip ratio 0.88 (0.08) and systolic blood pressure 129 (24) mmHg. For all these measurements the lowest values occurred in the normo-tolerant group while the highest was amongst participants with screen-detected diabetics. The general characteristics of the participants are summarised in Table 1.

### Biological profile

The median percentage of visceral fat in the overall sample was 10% and ranged from 9% in normo-tolerant individuals to 12.5% in screen-detected diabetes (p = 0.0008). Similarly the median percentage of body fat was 42.2% and ranged from 41.2% in the normo-tolerant group to 49.4% in participants with screen-detected diabetes (p = 0.044). Median resting metabolic rates were similar across all glucose tolerance groups (p = 0.146). The mean values (SD) for other biochemical measurements were 6.0% (1.2) for HbA1c, 5.5 (2.2) mmol/l for fasting glucose, 5.3 (1.1) mmol/l for total cholesterol, 1.3 (0.3) mmol/l for HDL cholesterol, and 3.3 (1.0) for measured LDL cholesterol. A median value of 7.6 U/l and 1.2 mmol/l was obtained for fasting insulin and triglycerides respectively and as expected there was a significant difference across the various glucose tolerance statuses (all p < 0.027), (Table 1). Median U-CRP, GGT and ALT also varied between the different groups (all p < 0.023), while values for cotinine and AST did not (both p > 0.191).

### Haematological profile

The full blood count parameters across all groups of participants were within the normal range. The median white cell count was 7.45 × 10^9^/l and the platelet count was 270 × 10^9^/l. The distribution of white cells was similar across all groups however the percentage of monocytes was lowest among screen-detected diabetics (p = 0.001). Although average haemoglobin, haematocrit and red cell measurements were within normal ranges, differences in the distribution across glucose tolerance statuses were apparent for mean cell volume (p = 0.019) mostly driven by high values in the known diabetics group (p = 0.019).

### Activation antigen expression on circulating monocytes and neutrophils

The median percentage of monocytes and neutrophils expressing the activation antigen CD69 was significantly different across glucose tolerance groups, being higher in known diabetics (26.2%), followed by the prediabetic group (16.9%), then screen detected diabetes (10.6%) and normo-tolerant (14.1%) (p = <0.016). An equivalent pattern was observed for neutrophils (p = 0.002) and the percentage of monocytes expressing CD142 (tissue factor), (p = 0.009). The percentage of neutrophils expressing tissue factor was similar across all groups of participants (p = 0.903), and there was no significant difference in the mean fluorescent intensity (MFI) of expression for any of the measured antigens across all glucose tolerance groups (Table 2).

Platelet activation as measured by the percentage of monocytes and neutrophils (PMA’s and PNA’s) expressing CD42b was significantly different across glucose tolerance groups, being highest in known diabetes followed by prediabetes, and lowest in screen-detected diabetes (p = <0.003), Table 2 and Fig. 1.

### Robust regression analysis

In robust linear regressions adjusted for age and gender, known diabetes was significantly and positively associated with the percentage of monocytes expressing CD69 [beta 11.06 (p-value = 0.016)], CD42b (PMAs) [19.51(0.003)], % of neutrophils expressing CD69 [14.19 (<0.0001)] and CD42b [17.7 (0.001)] Table 3. In similar robust regression models BMI was negatively associated with the percentage of monocytes expressing CD69 [beta −0.377 (p = 0.020)], CD42b [−0.441 (0.034)] and CD142 [−0.155 (0.021)]. The percentage of neutrophils expressing CD142 was also negatively associated with BMI [−0.155 (0.021)] while borderline associations were apparent with CD69 (p = 0.071) and CD42b (p = 0.056); Table 3. No significant associations were detected between markers of platelet, monocyte or neutrophil activation and gender, age, U-CRP levels and markers of glucose homeostasis.

## Discussion

Findings from the current study showed a differential distribution of levels of monocyte/neutrophil activation and platelet leucocyte aggregates by glucose tolerance status, mostly driven by high levels in participants with known diabetes, and to some extent low levels in those with screen-detected diabetes. These findings were further confirmed in robust regressions, whereby, known diabetes was significantly and positively associated with the percentage of monocytes or neutrophils expressing CD69 or CD142.

Although no association between U-CRP and the activation of platelets, monocytes or neutrophils could be detected in this study, elevated percentages of pro-inflammatory monocytes and neutrophils expressing both tissue factor (TF) and CD69 were observed in known diabetic participants. The alteration of activation antigens on cells of the innate immune system in type 2 diabetics has been previously described^26^,^27^,^28^ while others have reported that the activation of these cells together with the pro-inflammatory environment are linked to the progression of the disease and the onset of complications such as cardiovascular diseases^29^,^30^.

Tissue factor (CD142), which was increased on the surface of monocytes in this study, plays an important role in linking the process of inflammation with a pro-thrombotic environment. CD142 initiates the extrinsic pathway of coagulation by activating Factor VII and plays a role in stimulating the activation of platelets and leucocytes and the release of pro-inflammatory cytokines^29^,^31^. The expression of TF in type 2 diabetes however remains controversial. A study by Vambergue *et al*., reported no changes to monocyte tissue factor activity in T2D patients when compared to healthy individuals^32^. Notably however, this study was performed on a cohort of T2D patients who were not obese and in addition, the authors used an enzyme linked immunosorbent assay to measure soluble TF rather than cell surface expression^32^. The differing methodologies, BMI and glycaemic index could explain why the expression of tissue factor on monocytes in diabetes remains controversial.

The ability of granulocytes to synthesise and express Tissue Factor also remains controversial^33^,^34^. Neutrophils may acquire TF via direct interactions with activated monocytes^35^ and therefore could be implicated in the development of cardiovascular disease. The neutrophils in our study did not have significantly increased tissue factor expression in comparison to normal but did express CD69 which implies that they are activated and do contribute to the pro-inflammatory environment.

A number of researchers have further reported that untreated diabetic patients have dysfunctional monocytes and neutrophils which have defective activation, abnormal chemotaxis, phagocytosis and killing ability. Of note many of these functions are restored after treatment with glucose control agents^36^,^37^. Overall, although numbers are small, our results have shown that the activation status of monocytes and neutrophils were lower among the screen-detected diabetic participants and higher in the pre diabetic and known diabetic groups treated with at least metformin. This pattern could suggest that the persistently elevated levels of activation within the innate immune system eventually leads to immune exhaustion and dysfunction, a common finding in patients with newly diagnosed but untreated diabetes^36^,^37^,^38^. Treatment with drugs such as metformin restore immune function, however as the disease progresses the activation of monocytes and neutrophils increases leading to the formation of platelet leucocyte aggregates (PLAs) which are associated with the development of cardiovascular complications and thrombotic plaques^18^,^19^.

Platelet Monocyte Aggregates (PMA’s) are a robust measure of platelet activation and are also an indicator of vascular damage^13^. In agreement with our findings, several studies however, have failed to show an association between elevated PMAs and markers of glucose control^19^,^39^. Platelet leucocyte aggregates while increased in the prediabetic and known diabetic groups were lower in patients who had been newly diagnosed. This phenomenon seems to support the theory that the activation of platelets is not linked to glucose metabolism but rather the progression of the disease and the onset of vascular complications^19^,^40^. This theory remains controversial however, and a study by Patkό *et al*., reported a direct association between elevated levels of PMAs and fasting plasma glucose levels. Of note this was a small cohort of 14 T2D patients with no clinical signs of inflammation^15^. This could imply that the increased circulating levels of PMAs involve multifactorial mechanisms that act in synergy at a cellular level. These could be associated with both monocyte and platelet activation which initiate a cascade of signalling events which drive the formation of PMAs.

This was a cross-sectional study with no objective assessment of the presence of vascular complications to possibly explain some of our correlations. Longitudinal studies are therefore needed to probe the underlying mechanisms involving PMAs and increased monocyte-neutrophil interactions in T2D. There were an unequal number of participants across our glucose tolerance subgroups, and it is likely that the small number in some subgroups might have affected our capacity to uncover and characterise some of the associations.

The strengths of our study include the efforts to ensure reproducibility and standardization of flow cytometry based PLA measurement by following the guidelines recommended by the British International Committee of Haematology^24^. Artefactual platelet activation was avoided by minimising the time between sample collection and data acquisition, the omission of washing steps, mechanical stimulation due to centrifugation and the avoidance of buffers containing magnesium chloride and calcium chloride. In addition bias was avoided by only revealing the glycaemic status of the participants after the results had been processed and analysed.

In conclusion, this study has demonstrated increased levels of activated monocytes, neutrophils and platelets in patients with known diabetes which are not associated with markers of glucose metabolism. Platelet leucocyte aggregate formation increases as cardiovascular complications develop and therefore the monitoring of leucocyte activation and platelet leukocyte aggregates in type 2 diabetes may be crucial in the management and risk stratification of patients at risk of developing thrombotic complications.

## Additional Information

**How to cite this article:** Davison, G. M. *et al*. Platelet, monocyte and neutrophil activation and glucose tolerance in South African Mixed Ancestry individuals. *Sci. Rep.* **7**, 40329; doi: 10.1038/srep40329 (2017).

**Publisher's note:** Springer Nature remains neutral with regard to jurisdictional claims in published maps and institutional affiliations.

## Figures and Tables

**Table 1:** Characteristics of 206 participants according to glucose tolerance status.

	Overall	Normol	Pre-diabetes	Screen-detected Diabetes	Known Diabetes	p-value
N	206 (100)	136 (66.0)	39 (18.9)	18 (8.7)	13 (6.3)	
Female, *n* (%)	147 (71.4)	95 (69.8)	29 (74.4)	13 (72.2)	10 (76.9)	0.913
Smoking, n (%)	88 (42.9)	65 (48.1)	13 (33.3)	4 (22.2)	6 (46.1)	0.103
Age (years)	52.2 (14.0)	49.8 (14.0)	57.1 (14.4)	56.4 (12.2)	56.5 (7.2)	0.007
BMI, kg/m^2^	29.5 (8.1)	28.6 (8.6)	30.1 (7.3)	33.6 (5.0)	31.1 (6.4)	0.045
Waist-C, cm	95 (15)	93 (16)	98 (13)	107 (10)	102 (13)	0.0005
Hip-C, cm	108 (15)	107 (16)	109 (14)	111 (9)	110 (14)	0.405
Waist-to-hip ratio	0.88 (0.08)	0.87 (0.08)	0.90 (0.07)	0.96 (0.07)	0.92 (0.06)	<0.0001
Systolic BP, mmHg	129 (24)	124 (23)	136 (23)	146 (25)	129 (17)	0.0005
Diastolic BP, mmHg	84 (13)	83 (13)	84 (12)	89 (13)	87 (9)	0.222
Visceral fat, levels	10 [7–13]	9 [6–12]	10.5 [8.7–13.2]	12.5 [10.0–13.7]	12.0 [9.7–14.0]	0.0008
Body fat, %	42.2 [30.4–49.2]	41.2 [27.5–48.4]	44.3 [33.6–48.7]	49.4 [40.1–51.4]	45.9 [35.5–50.6]	0.044
RMR, Kcal	1469 [1324–1625]	1461 [1300–1601]	1460 [1378–1623]	1564 [1498–1622]	1542 [1416–1708]	0.146
HbA1c (%)	6.0 (1.2)	5.6 (0.4)	5.9 (0.6)	7.3 (1.2)	8.7 (2.5)	<0.0001
FBG, mmol/L	5.5 (2.2)	4.7 (0.5)	5.5 (0.7)	8.1 (2.6)	10.8 (4.6)	<0.0001
2-hour glucose, mmol/L	7.0 (3.3)	5.4 (1.3)	8.9 (1.4)	14.7 (3.7)	—	<0.0001
Fasting insulin, mIU/L	7.6 [5.0–10.9]	6.8 [4.2–9.5]	9.1 [6.3–13.9]	12.4 [8.1–20.1]	12.4 [8.1–20.1]	<0.0001
2-hour insulin, mIU/L	47.4 [24.7–79.4]	35.0 [17.8–63.6]	70.3 [48.2–112.4]	72.7 [44.9–88.6]	—	<0.0001
TC, mmol/L	5.3 (1.1)	5.3 (1.1)	5.1 (0.9)	6.0 (1.2)	5.6 (1.1)	0.027
HDL-C, mmol/L	1.3 (0.3)	1.3 (0.3)	1.3 (0.3)	1.1 (0.2)	1.0 (0.2)	0.010
LDL-C, mmol/L	3.3 (1.0)	3.3 (0.9)	3.2 (0.9)	4.0 (1.0)	3.7 (0.9)	0.009
Triglycerides, mmol/L	1.2 [0.9–1.7]	1.1 [0.8–1.6]	1.4 [0.9–1.8]	1.7 [1.4–2.3]	1.6 [1.2–2.5]	<0.0001
U-CRP, mg/L	4.0 [1.9–9.5]	3.6 [1.4–9.1]	3.9 [1.9–6.5]	7.3 [3.8–14.7]	8.7 [3.0–12.6]	0.023
Cotinine, ng/mL	10 [10–279]	10 [10–293]	10 [10–243]	10 [10–10]	10 [10–269]	0.191
γGT, U/L	31 [21–45]	30 [20–40]	27 [22–41]	61 [37–82]	31 [26–68]	0.002
AST, U/L	22 [19–26]	22 [19–25]	21 [18–25]	24 [21–32]	22 [15–30]	0.271
ALT, U/L	17 [13–23]	17 [13–22]	17 [13–21]	28 [20–36]	20 [13–27]	0.002
RBCx10^12/l	4.72 [4.36–5.00]	4.70 [4.34–5.01]	4.60 [4.32–4.90]	4.91 [4.58–5.26]	4.73 [4.38–4.97]	0.127
Haematocrit, L/L	41 [39–44]	42 [39–44]	41 [39–44]	41 [39–44]	41 [40–43]	0.936
Haemoglobin, g/dL	13.6 [12.8–14.4]	13.6 [12.8–14.4]	13.5 [12.7–14.2]	13.5 [13.0–14.1]	13.6 [13.0–14.1]	0.930
MCV, fl	89 [85–93]	89 [85–93]	89 [86–94]	84 [79–88]	90 [85–92]	0.019
MCH, pg	29 [28–30]	29 [28–31]	29 [28–31]	28 [26–29]	30 [29–30]	0.024
MCHC, g/dL	33 [32–33]	33 [32–33]	33 [32–33]	33 [32–33]	33 [32–33]	0.644
RDW, %	14.1 [13.4–14.8]	14.0 [13.5–14.8]	14.1 [13.3–14.8]	14.8 [14.0–15.3]	13.6 [13.0–13.8]	0.022
WCC x10^9/l	7.45 [6.30–9.30]	7.30 [6.10–9.30]	7.70 [6.50–9.10]	7.55 [6.87–9.05]	7.90 [7.50–10.70]	0.490
Lymphocytes, x10^9/l	2.20 [1.80–2.63]	2.20 [1.80–2.65]	2.20 [1.80–2.60]	2.30 [1.85–2.87]	2.30 [1.90–2.50]	0.772
Lymphocytes %	29.7 [23.6–35.9]	30.0 [23.3–36.5]	28.8 [24.6–34.9]	31.9 [25.0–36.0]	27.4 [21.1–32.7]	0.695
Monocytes, x10^9/l	0.50 [0.40–0.60]	0.50 [0.40–0.60]	0.40 [0.33–0.50]	0.40 [0.33–0.50]	0.50 [0.40–0.55]	0.151
Monocytes %	6.0 [5.1–7.4]	6.4 [5.4–7.7]	5.4 [4.9–6.5]	5.1 [4.4–5.9]	6.0 [5.1–6.9]	0.001
Neutrophils, x10^9/l	4.60 [3.60–5.80]	4.30 [3.30–5.67]	4.80 [3.82–6.12]	4.90 [4.00–5.67]	4.90 [4.00–5.67]	0.257
Neutrophils %	60.5 [54.7–66.6]	59.8 [52.9–66.4]	63.1 [56.2–66.5]	59.4 [56.0–67.5]	62.7 [60.0–71.4]	0.189
Basophils, x10^9/l	0.01 [0.01–0.02]	0.01 [0.01–0.01]	0.01 [0.01–0.01]	0.01 [0.01–0.01]	0.01 [0.01–0.04]	0.356
Basophils %	0.4 [0.3–0.6]	0.5 [0.3–0.6]	0.4 [0.3–0.5]	0.4 [0.3–0.5]	0.3 [0.3–0.7]	0.224
Eosinophils, x10^9/l	0.13 [0.10–0.20]	0.19 [0.10–0.30]	0.10 [0.10–0.20]	0.14 [0.10–0.20]	0.10 [0.10–0.20]	0.096
Eosinophil %	1.0 [1.1–3.1]	2.2 [1.4–3.4]	1.7 [1.0–2.4]	2.0 [1.1–2.5]	1.1 [0.8–2.2]	0.023
Platelets, x10^9/l	270.0 [225.0–315.5]	271.0 [226.0–318.2]	278.0 [237.7–322.5]	251.0 [236.2–288.7]	2230 [200.0–318.0]	0.527

γGT, γ-Glutamyltransferase; ALT, Alanine aminotransferase; AST, Aspartate transaminase; BMI, Body mass index; BP, blood pressure; FBG, Fasting blood glucose; Hip-C, Hip circumference; MCH, mean cell haemoglobin; MCHC, mean corpuscular haemoglobin concentration; HDL-C, High density lipoprotein cholesterol; LDL-C, Low density lipoprotein cholesterol; MCV, mean cell volume; RBC, Red cell count; RDW, Red cell distribution width; RMR, Resting metabolic rate; TC, Total cholesterol U-CRP, ultra-sensitive C-reactive protein; Waist-C, Waist circumference; WCC, White cell count.

**Table 2:** Activation antigen expression according to glucose tolerance status.

Variables	Overall	Normoglycemia	Pre-diabetes	Screen-detected Diabetes	Known Diabetes	p-value
Markers of Monocyte activation						
%CD69 expression	14.7 [5.99–27.8]	14.1 [5.0–25.1]	16.9 [7.2–38.6]	10.6 [6.9–14.8]	26.2 [14.2–37.8]	0.016
CD69 MFI	2.9 [2.4–3.8]	2.9 [2.3–3.7]	3.2 [2.5–4.8]	2.8 [1.8–3.7]	2.9 [2.6–3.5]	0.240
%CD42b expression	36.9 [17.8–52.9]	35.7 [18.8–49.5]	44.6 [18.7–61.7]	17.6 [11.7–43.6]	65.1 [37.2–76.2]	0.004
CD42 MFI	5.7 [4.3–7.6]	5.6 [4.3–7.6]	6.0 [4.6–7.6]	5.7 [4.3–8.7]	6.1 [4.6–7.3]	0.980
%CD142 expression	5.9 [2.9–12.3]	5.5 [2.5–10.4]	7.2 [4.5–17.1]	3.5 [2.2–7.7]	12.0 [5.2–17.1]	0.009
CD142 MFI	2.4 [2.1–4.4]	2.3 [2.1–4.3]	2.7 [2.1–5.0]	3.1 [2.0–7.1]	2.3 [2.1–2.4]	0.713
Markers of Neutrophil activation						
%CD69 expression	5.5 [2.1–14.1]	5.0 [1.9–12.0]	7.6 [2.8–14.6]	2.1 [1.2–6.4]	20.8 [10.1–24.0]	0.002
CD69 MFI	2.7 [2.3–3.3]	2.7 [2.2–3.4]	2.7 [2.4–3.2]	2.7 [2.2–3.7]	2.4 [2.0–3.0]	0.556
%CD42b expression	22.0 [6.2–38.5]	21.5 [6.2–37.2]	26.0 [7.5–40.8]	8.9 [4.1–23.9]	50.4 [23.8–53.0]	0.003
CD42b MFI	4.5 [3.4–5.8]	4.4 [3.4–5.5]	5.0 [3.8–6.6]	4.5 [3.7–5.7]	4.3 [3.6–5.4]	0.493
%CD142 expression	12.5 [5.7–12.5]	5.5 [2.6–12.1]	5.4 [3.5–15.2]	7.4 [1.9–10.7]	7.5 [3.7–12.2]	0.903
CD142 MFI	3.7 [2.3–6.5]	3.7 [2.3–6.3]	4.2 [2.7–6.7]	5.2 [3.6–6.5]	2.6 [2.3–3.3]	0.124

**Table 3:** Regression beta coefficients (p-values) from multiple robust linear models for the prediction of markers of platelet activation and platelet leucocytes aggregates, accounting for the potential effect of sex and age, diabetes and adiposity.

Predictors	Markers of Monocyte activation						Markers of Neutrophil activation					
	%CD69	CD69 MFI	%CD42	CD42 MFI	%CD142	CD142 MFI	%CD69	CD69 MFI	%CD42	CD42 MFI	%CD142	CD142 MFI
Sex (male)	0.427 (0.876)	−0.101 (0.565)	3.133 (0.367)	−0.304 (0.426)	−0.132 (0.903)	−0.002 (0.991)	0.720 (0.554)	−0.140 (0.300)	2.127 (0.547)	−0.025 (0.921)	−0.131 (0.903)	−0.663 (0.069)
Smoking	2.841 (0.273)	−0.032 (0.851)	2.736 (0.433)	0.029 (0.936)	1.770 (0.161)	−0.079 (0.573)	1.499 (0.189)	0.037 (0.782)	2.490 (0.455)	0.025 (0.918)	1.770 (0.161)	−0.366 (0.379)
Age	0.009 (0.917)	−0.009 (0.102)	0.110 (0.329)	−0.009 (0.471)	0.057 (0.102)	0.003 (0.546)	−0.007 (0.951)	0.001 (0.749)	0.079 (0.489)	0.006 (0.434)	0.057 (0.102)	−0.019 (0.112)
BMI	−0.377 (0.020)	−0.001 (0.947)	−0.441 (0.034)	−0.025 (0.288)	−0.155 (0.021)	0.003 (0.704)	−0.171 (0.071)	−0.006 (0.472)	−0.375 (0.056)	−0.007 (0.689)	−0.155 (0.021)	0.031 (0.292)
U-CRP	0.086 (0.207)	0.003 (0.609)	0.071 (0.400)	0.006 (0.512)	−0.008 (0.842)	−0.005 (0.217)	0.024 (0.532)	<−0.0001 (0.994)	−0.018 (0.800)	−0.003 (0.802)	−0.008 (0.842)	0.015 (0.130)
Glucose tolerance status												
Normotolerent	Reference	Reference	Reference	Reference	Reference	Reference	Reference	Reference	Reference	Reference	Reference	Reference
Prediabetes	4.115 (0.169)	0.460 (0.034)	4.312 (0.300)	0.594 (0.228)	1.203 (0.372)	0.112 (0.530)	1.513 (0.279)	0.146 (0.386)	2.153 (0.529)	0.470 (0.141)	1.203 (0.372)	0.254 (0.580)
New Diabetes	−5.884 (0.157)	−0.023 (0.926)	−10.450 (0.066)	0.400 (0.557)	−1.902 (0.289)	0.140 (0.584)	−2.178 (0.248)	−0.231 (0.355)	−8.412 (0.069)	0.133 (0.754)	−1.902 (0.289)	1.405 (0.019)
Known diabetes	11.061 (0.016)	0.165 (0.605)	19.513 (0.003)	0.266 (0.717)	3.121 (0.138)	−0.089 (0.726)	14.195 (<0.0001)	−0.087 (0.721)	17.697 (0.001)	0.169 (0.725)	3.121 (0.138)	−0.758 (0.259)
HbA1c	0.431 (0.784)	0.033 (0.683)	0.602 (0.758)	0.089 (0.688)	−0.119 (0.793)	0.016 (0.779)	−0.055 (0.927)	−0.007 (0.924)	0.050 (0.971)	0.095 (0.339)	−0.119 (0.793)	0.052 (0.766)
FBG	0.685 (0.275)	0.036 (0.300)	1.353 (0.065)	0.080 (0.273)	0.108 (0.645)	−0.001 (0.986)	0.541 (0.067)	−0.012 (0.659)	0.952 (0.127)	0.054 (0.288)	0.109 (0.645)	−0.052 (0.578)
Fasting insulin	−0.011 (0.964)	−0.004 (0.251)	−0.122 (0.714)	−0.004 (0.596)	−0.031 (0.176)	−0.0001 (0.996)	−0.030 (0.741)	−0.003 (0.347)	−0.089 (0.604)	−0.0004 (0.990)	−0.031 (0.176)	−0.002 (0.969)

For regression using glucose tolerance status as predictor, the subgroup of participants with normal glucose tolerance (normotolerant) served as reference group for all comparisons. This means beta coefficients for other glucose tolerance subgroups represents the absolute change in the level of each marker of interest, from the mean level in the subgroup of participants with normal glucose tolerance.
